# Evaluation of the Cerebrospinal Fluid (CSF)-Truenat Assay: A Novel Chip-Based Test in the Diagnosis and Management of Tubercular Meningitis at a Tertiary Care Hospital

**DOI:** 10.7759/cureus.74522

**Published:** 2024-11-26

**Authors:** Subhash Nanda, Manish K Bansal, Priya Singh, Ashish K Shrivastav, Manoj K Malav, Chandra Prakash

**Affiliations:** 1 Medicine, Sarojini Naidu Medical College, Agra, IND; 2 Internal Medicine, Sarojini Naidu Medical College, Agra, IND; 3 Neurology, Sarojini Naidu Medical College, Agra, IND; 4 Emergency Medicine, Sarojini Naidu Medical College, Agra, IND

**Keywords:** cbnaat, cerebrospinal fluid, gene xpert, mycobacterium tuberculosis, truenat, tubercular meningitis

## Abstract

Introduction: Tuberculosis (TB) continues to be a major health concern that has a significant impact on morbidity and mortality worldwide. Tubercular meningitis (TBM) may be fatal due to its severe neurological outcomes if not diagnosed and treated promptly. The newer molecular diagnostic techniques have brought significant advancements in the detection of *Mycobacterium tuberculosis* (MTB). One such test is the cerebrospinal fluid (CSF)-Truenat assay which offers several advantages over traditional methods and provides results within a few hours. This is crucial for timely intervention and can further improve patient outcomes. We have evaluated its diagnostic accuracy, its utility, and its usefulness in treatment decisions.

Methods: A cross-sectional study was conducted on 150 patients. The CSF samples were analyzed by both cartridge-based nucleic acid amplification test (CBNAAT) and Truenat (Molbio Diagnostics Private Limited, Verna, Goa, India). Brain imaging was also performed. Patients were divided into four groups, i.e., definite, probable, possible, and non-TBM. Truenat-positive cases were considered definite, and their efficacy and clinical utility for diagnosing and managing TBM were compared with CBNAAT.

Results: A comparative analysis between CBNAAT and Truenat showed concordance for positive results. But it also identified some discrepancies, particularly in cases where CBNAAT was positive and Truenat was negative. Overall, Truenat demonstrated strong diagnostic performance with a sensitivity of 83.75%, a specificity of 88.57%, and overall accuracy of 86% as compared to CBNAAT.

Conclusion: The study highlighted the role of Truenat in improving diagnostic accuracy and guiding cost-effective treatment strategies for TBM as compared to CBNAAT. As molecular tests alone cannot detect bacilli in most cases, a combination of clinical, microbiological, and radiological parameters is also obligatory for diagnosing TBM.

## Introduction

Tuberculosis (TB) continues to be a major public health concern since it has a significant impact on morbidity and mortality rates across the globe. Low- and middle-income nations are the worst hit by the disease. India has the highest TB burden in the world, with more than 24 lakh cases reported in the year 2022 [1]. Tubercular meningitis (TBM) accounts for around 5%-10% of extra-pulmonary TB cases and is associated with a high mortality rate of 40%-60%. Nearly half of the remaining patients are affected by neurological sequelae [2,3]. Early and timely interventions are thus mandated for the diagnosis and management of the disease. Analysis of cerebrospinal fluid (CSF), its microscopy, and culture are a few conventional diagnostic methods for TBM. Cerebrospinal fluid smear microscopy has low sensitivity. On the contrary, the culture of *Mycobacterium tuberculosis* (MTB) from CSF is more sensitive, but it takes several weeks to yield results. This delay can be quite detrimental, as appropriate anti-tubercular therapy must be initiated at the earliest to reduce morbidity and mortality associated with TBM [4]. These limitations entail the need for a quicker and more reliable diagnostic tool for TBM.

The WHO endorses the use of the cartridge-based nucleic acid amplification test (CBNAAT, Cepheid GeneXpert System, Cepheid, Sunnyvale, CA, USA) system for the diagnosis of extrapulmonary TB; CBNAAT testing requires specialized equipment and trained personnel and is more expensive, thereby limiting its use in resource-constrained settings. An Indian company, Molbio Diagnostics Private Limited (Verna, Goa, India), has developed a Truenat assay, which is a real-time polymerase chain reaction (PCR)-based test. The Truenat MTB assay is an automated, battery-operated, portable device that is designed to be used at sites with minimal infrastructure, like peripheral health centers or mobile vans [5]. It has a limit of detection of 30 colony-forming units (cfu)/ml. The assay identifies the presence of the ribonucleoside-diphosphate reductase gene (nrdZ gene), which codes for ribonucleoside-diphosphate reductase adenosyl cobalamin-dependent protein. Furthermore, it also detects the IS6110 gene sequence. Extraction of DNA and detection of MTB takes approximately 40 minutes. If results are positive, a sample of already extracted DNA may be loaded onto a Truenat MTB Rif chip and can be analyzed for rifampicin resistance [5]. Truenat has been evaluated for pulmonary samples and has shown higher sensitivity and specificity. Till now, less research has been done on CSF samples using Truenat in adult patients with suspected TBM.

There is enormous data available on the utility of CBNAAT in diagnosing TBM. However, the available data on Truenat testing are quite limited. This further necessitates the dire need for further study to evaluate the diagnostic accuracy of CSF-Truenat assays. Hence, this study was conducted to evaluate the utility and cost-effectiveness of the CSF-Truenat assay in the diagnosis and management of TBM.

## Materials and methods

A hospital-based, observational, cross-sectional study was conducted in the postgraduate department of Medicine at Sarojini Naidu Medical College, a tertiary care hospital in Agra, India from October 2022 to June 2024. A total of 150 patients over 14 years of age exhibiting clinical signs and symptoms suggestive of TBM were enrolled after obtaining proper consent. Participants were given the opportunity to ask questions and withdraw from the study at any time. The risks, benefits, and purposes of the study were clearly explained to the participants in their own language. Ethical approval was received from the Institutional Ethics Committee (SNMC/IEC/2024/305) of Sarojini Naidu Medical College. Confidentiality of the participants was maintained throughout the study. Patients having evidence of meningitis apart from TB, end-stage renal or liver disease, severe sepsis, pregnancy, age below 14 years, and having contraindications for lumbar puncture or refusal for consent were excluded from the study.

Enrolled patients were initially assessed through detailed clinical history and neurological examinations. Laboratory data, including CBC, blood sugar levels, kidney and liver function tests, thyroid function tests, and viral markers (hepatitis B, hepatitis C, and HIV), were collected. Radiological investigations, including chest X-ray, contrast-enhanced CT scan of the brain, and contrast-enhanced MRI of the brain (if required), were done. The CSF samples (4 ml) were obtained using a sterile technique; 2 ml of CSF was sent for cytological and biochemical analysis, and the remaining 2 ml was processed for CBNAAT and Truenat assays. Rifampicin resistance was tested by both methods. Truenat Auto Prep version 2 (Molbio Diagnostics Private Limited) was used to extract and purify nucleic acid. It was then loaded into a PCR analyzer. If MTB was detected in any sample, a portion of extracted DNA was tested for Rif resistance using the Truenat MTB-Rif Dx chip (Molbio Diagnostics Private Limited) [5]. Truenat-positive cases were considered definite, and their efficacy and clinical utility were compared with CBNAAT, which was considered the gold standard in this study.

Diagnosis of TBM was based on the standard case definition by the Working Committee Consensus 2010 [6]. According to the scoring, patients were categorized into four groups, i.e., definite, possible, probable, and non-TBM.

## Results

A total of 150 patients having clinical signs and symptoms suggestive of TBM were included in the study. It was found that most of the patients (46.7%) were in the age group of 15 to 35 years; 39.3% of patients were in the age group of 36 to 55 years, and 14% were older than 55 years of age. There were 104 male subjects, constituting 69.3% of the total participants, and 46 female subjects, accounting for 30.7% of the total participants.

Seventy-five cases tested positive in Truenat, representing 50% of the sample, and the rest tested negative on Truenat, whereas 80 cases tested positive in CBNAAT, accounting for 53.3% of total participants, and 70 tested negative (46.7%). Table 1 and Table 2 show the distribution of study subjects according to Truenat and CBNAAT results, respectively.

**Table 1:** Distribution of the study subjects according to Truenat results TBM: tubercular meningitis

Truenat	Category	Frequency	Percentage (%)
Positive	Definite	75	50
Negative	Probable	49	32.7
	Possible	23	15.3
	Non-TBM	3	2

**Table 2:** Distribution of study subjects according to CBNAAT CBNAAT: cartridge-based nucleic acid amplification tests; TBM: tubercular meningitis

CBNAAT	Category	Frequency	Percentage (%)
Positive	Definite	80	53.3
Negative	Probable	46	30.7
	Possible	21	14
	Non-TBM	3	2

Truenat detected rifampicin resistance in 13 cases among 75 positive cases, whereas CBNAAT detected rifampicin resistance in 13 among 80 positive cases. Table 3 and Table 4 represent rifampicin resistance among definitive cases by Truenat and CBNAAT, respectively.

**Table 3:** Rifampicin resistance detected by Truenat

Rifampicin resistance	Frequency	Percentage (%)
Present	13	17.3
Absent	62	82.7

**Table 4:** Rifampicin resistance detected by CBNAAT CBNAAT: cartridge-based nucleic acid amplification tests

Rifampicin resistance	Frequency	Percentage (%)
Present	13	16.3
Absent	67	83.7

Table 5 represents the association between CBNAAT and Truenat findings. Sixty-seven cases were detected positive by both tests. However, in 13 cases, CBNAAT was positive while Truenat was negative. Sixty-two cases were detected negative by both tests. Eight cases were negative by CBNAAT but positive by Truenat.

**Table 5:** Relationship of CBNAAT findings with Truenat findings CBNAAT: cartridge-based nucleic acid amplification tests

		Truenat	
		Positive	Negative
CBNAAT	Positive	67	13
	Negative	8	62

Truenat showed a sensitivity of 83.75% and a specificity of 88.57%. The positive predictive value (PPV) for Truenat was reported as 89.33%, and the negative predictive value (NPV) was 82.67%. The overall accuracy of Truenat was reported as 86% with a 95% CI from 79.40% to 91.12%. Table 6 represents the diagnostic accuracy of Truenat as compared to CBNAAT.

**Table 6:** Diagnostic accuracy of Truenat in comparison to CBNAAT as the gold standard CBNAAT: cartridge-based nucleic acid amplification tests

Statistic	Value	95% CI
Sensitivity	83.75%	73.82% to 91.05%
Specificity	88.57%	78.72% to 94.93%
Positive predictive value	89.33%	81.28% to 94.18%
Negative predictive value	82.67%	74.22% to 88.76%
Accuracy	86%	79.40% to 91.12%

## Discussion

Tubercular meningitis is a type of extra-pulmonary TB and has the potential to be lethal. Tubercular meningitis progresses quite rapidly, leading to severe complications like hydrocephalus, cerebral infarction, and cranial nerve palsies. Improved patient outcomes and efficient management depend on early and accurate diagnosis. The present study delves into the function of CSF-Truenat assays in the clinical diagnosis and treatment of individuals with TBM. It also highlights its cost-effectiveness and impact on improving diagnostic processes and patient care in a high-stakes healthcare environment.

In our study, all those cases that were Truenat-positive were considered definite. It included 75 cases, which represents 50% of the total study population. The probable category, which comprised cases that were likely to have the condition but were lacking in absolute confirmation, included 49 subjects, accounting for 32.6% of the total cases. The possible category comprised 23 (15.3%) subjects. Lastly, the non-TBM category constituted three (2%) subjects.

This distribution indicates that half of the study population was diagnosed with a positive Truenat, while the other half was detected as negative. This balanced distribution provides a clear comparative baseline for further analysis of factors associated with Truenat test results. A study conducted on sputum samples by Nikam et al. (2013) [7] reported comparable findings, with 191 out of 226 patients diagnosed with definite and probable TBM, while the remaining 35 cases were classified as non-TBM.

Out of the total cases detected positive by Truenat, 62 cases (82.7%) were negative for rifampicin resistance. It indicates that the majority of the cases did not exhibit resistance to the antibiotic. Thirteen cases (17.3%) were having rifampicin resistance. This group highlights the presence of antibiotic resistance and the importance of early detection in the management of disease. In a study conducted by Meaza et al. (2021) [8] on sputum samples in 200 adult participants, out of 25 confirmed TB cases, only one case exhibited rifampicin resistance. This is crucial for understanding the prevalence of drug resistance, which can help in selecting the most appropriate treatment strategy for managing the condition effectively.

As compared to CBNAAT, Truenat showed a sensitivity of 83.75% and a specificity of 88.57%. The PPV for Truenat was reported as 89.33%, and NPV was 82.67%. Overall, the accuracy of Truenat is reported to be 86%. These values indicate Truenat's strong performance as a diagnostic tool for the disease and are comparable to CBNAAT. Hence, establishing its reliability and effectiveness in the clinical setting. In a study conducted by Sharma et al. (2021) [9] on CSF samples, Truenat MTB Plus and GeneXpert Ultra were used for the detection of MTB. The study included 148 CSF specimens. The overall sensitivity of Truenat in diagnosing TBM was 78.7%, and for diagnosing definite TBM, it was 85.5%. The overall sensitivity of GeneXpert Ultra in diagnosing TBM was 67.7%, and in diagnosing definite TBM, it was 96%.

In this study, some discrepancies were noted in the cases of discordant results. There were 13 cases that were detected positive by CBNAAT and negative by Truenat (false negatives), which can lead to delayed treatment, further worsening the patient's outcome. There were eight cases that were detected positive by Truenat and negative by CBNAAT (false positives), which can result in an unnecessary burden on patients and the health care system. So, there is a need for further studies to address this issue. 

However, there were some limitations in this study. The sample size was 150, which might affect the applicability of this study's results to a larger population. The study only highlighted rifampicin resistance among definitive TBM cases, but it did not tell anything about other forms of drug resistance or the potential of multi-drug-resistant TBM. Other contributing factors, such as comorbidities and treatment compliance, were not considered in this study. There was the loss to follow-up bias, so the outcome in these individuals could not be properly assessed.

## Conclusions

The comprehensive analysis of CBNAAT and Truenat showed a strong diagnostic concordance for positive results. However, some discrepancies were noted in the cases of discordant results. Truenat is a portable device that is battery-operated. It is cost-effective, yields results in less time, and shows similar results as compared to CBNAAT. It has a farther reach and can be utilized in low-resource settings. However, it’s still in the infancy stage, and continued research on CSF samples is needed to prove its efficacy and to generalize results in larger populations. The diagnosis of TBM requires a combination of clinical, microbiological, and radiological parameters, as molecular tests alone cannot detect bacilli in most cases.
